# Measurement properties, feasibility and clinical utility of the Doloplus-2 pain scale in older adults with cognitive impairment: a systematic review

**DOI:** 10.1186/s12877-017-0643-9

**Published:** 2017-11-02

**Authors:** Hanne Marie Rostad, Inger Utne, Ellen Karine Grov, Martine Puts, Liv Halvorsrud

**Affiliations:** 1 Department of Nursing and Health Promotion, Faculty of Health Sciences, Oslo, Norway; 2 Akershus University College of Applied Sciences, P.O. Box 4, St. Olavs Plass, N-0130 Oslo, Norway; 3 Lawrence S Bloomberg Faculty of Nursing, University of Toronto, Toronto, Canada

**Keywords:** Cognitive impairment, Dementia, Doloplus-2, Pain, Older adults, Systematic review

## Abstract

**Background:**

The Doloplus-2 is a pain assessment scale for assessing pain in older adults with cognitive impairment. It is used in clinical practice and research. However, evidence for its measurement properties, feasibility and clinical utility remain incomplete. This systematic review synthesizes previous research on the measurement properties, feasibility and clinical utility of the scale.

**Method:**

We conducted a systematic search in three databases (CINAHL, Medline and PsycINFO) for studies published in English, French, German, Dutch/Flemish or a Scandinavian language between 1990 and April 2017. We also reviewed the Doloplus-2 homepage and reference lists of included studies to supplement our search. Two reviewers independently reviewed titles and abstracts and performed the quality assessment and data abstraction.

**Results:**

A total of 24 studies were included in this systematic review. The quality of the studies varied, but many lacked sufficient detail about the samples and response rates. The Doloplus-2 has been studied using diverse samples in a variety of settings; most study participants were in long-term care and in people with dementia. Sixteen studies addressed various aspects of the scale’s feasibility and clinical utility, but their results are limited and inconsistent across settings and samples. Support for the scale’s reliability, validity and responsiveness varied widely across the studies. Generally, the reliability coefficients reached acceptable benchmarks, but the evidence for different aspects of the scale’s validity and responsiveness was incomplete.

**Conclusion:**

Additional high-quality studies are warranted to determine in which populations of older adults with cognitive impairment the Doloplus-2 is reliable, valid and feasible. The ability of the Doloplus-2 to meaningfully quantify pain, measure treatment response and improve patient outcomes also needs further investigation.

**Trial registration:**

PROSPERO reg. no.: CRD42016049697 registered 20. Oct. 2016.

**Electronic supplementary material:**

The online version of this article (10.1186/s12877-017-0643-9) contains supplementary material, which is available to authorized users.

## Background

Cognitive impairment is increasing globally [1], as is the global population over 60 years old [2]. Pain is a well-documented, very prevalent issue in older adults with cognitive impairment, who often suffer from conditions like musculoskeletal disorders, malignancy, gastrointestinal and cardiac conditions [3–5]. It is estimated that at least 50% of older adults with cognitive impairment residing in long-term care (LTC) facilities have pain on a regular basis [6, 7].

Pain assessment is essential for adequate pain management [7, 8], but assessing pain in older adults with cognitive impairment remains a challenging issue due to impaired memory, changes in cognitive processing, and a reduced ability or inability to communicate verbally [7, 9]. Thus, caregivers may need alternative methods to obtain information about the person’s pain. When older adults with cognitive impairment cannot report pain themselves, the next best option – the so-called ‘silver standard’ – is assessment by the person who is most familiar with the patient’s everyday life [10]. However, previous research has reported that pain assessment in older adults with cognitive impairment often depends on a health care provider’s (HCP) subjective impression and occasionally appears to be mere guesswork [11, 12]. Therefore, in clinical practice, it may be useful for HCPs to use pain assessment tools that account for the population’s distinctive characteristics. However, pain assessment tools are used infrequently, which may contribute to the fact that un(der)managed pain remains a major problem in this population [6, 13, 14]. Furthermore, there is limited evidence regarding the measurement properties, feasibility and clinical utility of pain assessment tools for older adults with cognitive impairment. Currently, no one particular tool is recommended [9, 15, 16]. However, a 2014 meta-review that reviewed 28 tools developed specifically for pain assessment in people with dementia identified the Doloplus-2 pain scale as one of the better tools currently available [9].

The Doloplus-2 is based on the Doloplus, which was developed by Wary et al. in 1993 [17]. The Doloplus was based on a tool that used behaviour to assess pain in children with neoplastic disease (the Douleur Enfant Gustave Roussy scale). The Doloplus assessed pain in older people with verbal communication difficulties by assessing their behaviour using three subscales: somatic, psychomotor and psychosocial reactions to pain. Each subscale included five items (for a total of 15 items), and each item received a score of 0, 1 or 2 [18]. In 1994, a network of geriatricians from Switzerland and France began developing the Doloplus-2, based on the Doloplus. The Doloplus-2 has the same three subscales, but the total number of items was reduced to ten:

1. Somatic reaction to pain includes five items: ‘somatic complaints’, ‘protective body postures adopted at rest’, ‘protection of sore areas’, ‘expression’ and ‘sleep pattern’.
2. Psychomotor reaction to pain includes two items: ‘washing and/or dressing’ and ‘mobility’.
3. Psychosocial reaction to pain includes three items: ‘communication’, ‘social life’ and ‘behaviour problems’.

The ten items on the Doloplus-2 are scored from 0 to 3; higher scores represent more intense pain [19]. The total score can range from 0 to 30. The score for the somatic reactions subscale ranges from 0 to 15, the psychomotor reaction subscale ranges from 0 to 6, and the psychosocial subscale ranges from 0 to 9. If the rater considers an item inappropriate, the item is not scored. A combined score of 5 or higher suggests the presence of pain [19].

The Doloplus-2 covers most of the pain behaviour categories recommended in the American Geriatric Society’s guidelines for ‘The management of persistent pain in older persons’ [20]; only ‘change in mental status’ is missing. The Dolopuls-2 includes the categories ‘facial expression’, ‘verbalizations/vocalization’, ‘body movements’, ‘changes in interpersonal interactions’ and ‘changes in activity patterns or routines’. The Doloplus-2 indicates a progression of pain rather than pain experienced in a specific moment [16]. An HCP (e.g. physician, registered nurse, nursing assistant) who knows the patient well should score the Doloplus-2. According to the developers, a trained HCP can complete the scale in approximately five minutes [17]. The Doloplus-2 was officially validated in 1999 and was published in English in 2001 [17, 19]. The tool has since been translated into many different languages [21–24].

Several reviews of pain assessment tools for older adults with cognitive impairment have been published, including a meta review [9]. Some of these include the Doloplus-2 [15, 16, 25–27]. However, more studies on the Doloplus-2 have been published since the last systematic review in 2012 (these reviewers conducted a systematic search up to 2010) [26]. The Doloplus-2 is one of the more extensively tested tools for pain assessment [9, 15], and it has been identified as one of the most promising tools for pain assessment in older adults with cognitive impairment [9]. Furthermore, the scale is used in clinical practices and research across the world. For this reason, this review focuses solely on the Doloplus-2. It seeks to thoroughly examine the scale’s feasibility, clinical utility and measurement properties when used to assess pain in older adults as this evidence remains incomplete. A feasible, useful and accurate scale is essential to ensure that older adults in pain are correctly identified as such, consistently and over time. Furthermore, for a pain scale to guide pain management decisions and support efficient evaluations, it must be actionable and easy to interpret, and it cannot take so many resources that it disrupts clinical care. Therefore, this systematic review examines the feasibility, clinical utility and measurement properties of the Doloplus-2 scale when used to assess pain in older adults with cognitive impairment.

## Method

This systematic review was prospectively registered with PROSPERO under reg. no. CRD42016049697. The PRISMA guidelines for reporting on systematic reviews were followed. Due to the clinical, methodological and statistical heterogeneity of the included studies, a descriptive approach was adopted in the research synthesis.

### Data sources and search strategy

A systematic search was conducted in CINAHL (March 2016), Medline (August 2016) and PsycINFO (September 2016) in collaboration with a research librarian. The search strategy was formulated in CINAHL and adapted in Medline and PsycINFO, using keywords, Boolean operators and the database’s controlled vocabulary. The results were limited from 1990 to the dates the searches were performed (Additional file 1).

In addition to the systematic search, a search for the keyword ‘Doloplus’ was performed in the three databases (February 2017). In CINAHL, ‘all text’ was selected so that the entire article text was searched for the term ‘Doloplus’. Medline and PsycINFO do not have the ‘all text’ option for searching with keywords, so only titles and abstracts were searched for the keyword. The systematic and keyword searches in all three databases were saved immediately, and e-mail alerts were set up for every search. We received automatic e-mail notifications from all three databases whenever a new publication matching our search criteria (for the systematic or the keyword search) became available in the database. These monthly auto-alerts were reviewed until April 2017, and articles which met the inclusion criteria were included in this review.

In addition to the database searches, the list of previous publications (including publications from 1993 to 2008) provided on the Doloplus-2 online home page was reviewed. Articles which met the inclusion criteria were included.

### Eligibility criteria

A study was eligible for inclusion if it: i) used the Doloplus-2 to assess pain in cognitively impaired patients (any stage) aged 65 and older; ii) were published in English, French, German, Dutch/Flemish or a Scandinavian language. Studies in which the Doloplus-2 was described but not used were excluded, as were studies in which the scale was used to validate other observational pain assessment tools. Dissertations, editorials, guidelines and expert opinion papers were excluded as well. Literature reviews were also excluded since they do not contain original data.

### Process of study selection

The studies were selected in two steps. First, two reviewers independently screened the titles and abstracts to determine the studies’ eligibility for inclusion. Discrepancies and uncertainties were discussed by the reviewer team until a consensus was reached. In the second step, two reviewers independently assessed the full text of the articles for eligibility. The reference lists of the included articles were also reviewed for additional eligible studies to supplement the data sources previously described.

### Quality assessment

Two reviewers independently assessed the quality of the included studies using the Mixed Methods Appraisal Tool (MMAT) [28]. The 2011 version of the MMAT allows for the description and appraisal of the methodological quality of five types of studies: i) qualitative, ii) quantitative randomized controlled trials, iii) quantitative non-randomized, iv) quantitative descriptive, and v) mixed methods. Each type has its own set of quality criteria. The criteria are scored ‘yes’, ‘no’ or ‘can’t tell’, followed by comments. The MMAT’s inter-rater reliability is moderate to excellent [29]. Since this is the first systematic review of the Doloplus-2, we wanted to provide a comprehensive review of the scale, so no study was excluded based on the quality assessment.

### Data abstraction

All the reviewers used a standardized data abstraction sheet. Two reviewers independently abstracted information from the studies, including study objective, setting, sample characteristics, how the Doloplus-2 was administered and the results of the assessment, and clinical utility and feasibility data. Feasibility was defined as the time and resources required to collect and process the assessment, encompassing ease of use, the need for staff training, and the time required to complete the assessment [30]. Clinical utility was defined as ‘usefulness to clinical practice’: the scale’s usefulness in identifying pain and whether the result of the assessment could assist clinical decisions (e.g. administration of analgesics) [10]. Information about the Doloplus-2’s measurement properties was also abstracted. As a guide for abstracting data on measurement properties, we used the COSMIN (COnsensus-based Standards for the selection of health Measurement INstruments) international consensus on taxonomy, terminology, and definitions of measurement properties for health-related patient-reported outcomes [31]. Different authors propose various criteria for assigning strength of association to particular values, but we chose the guidelines for instrument reliability and precision suggested by Hahn et al. [32].

## Results

**Fig. 1:**
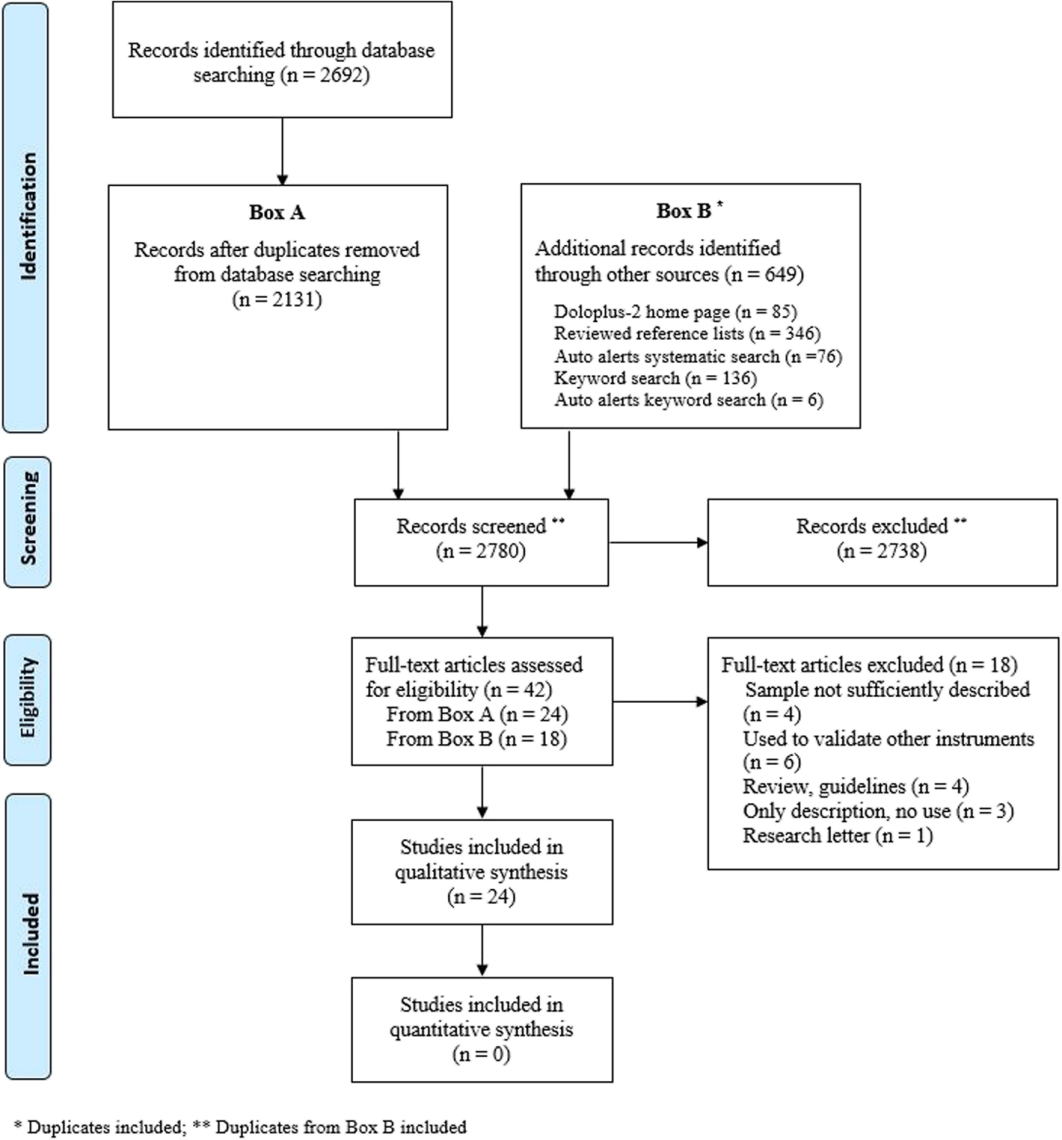
Flow diagram

A total of 2692 citations were initially identified for possible inclusion through the systematic search of the three databases. The citations were transferred into Endnote and duplicates were removed; 2131 unique citations remained (Box A). An additional 649 publications were identified through other sources (Box B). There were so many additional publications because the other sources were manually screened, and we did not have a reference system to remove duplicates or those already retrieved through the systematic search. In total, 2780 publications were screened. After the titles and abstracts were reviewed, 42 full-text studies were assessed for eligibility. We were unsure whether five articles met the eligibility criteria, and we attempted to contact the corresponding author via e-mail. For two of those, no e-mail address was found. Of the three authors contacted, two did not respond, and one provided sufficient information [33]. Consequently, four studies were excluded because we were unable to determine whether they fulfilled the eligibility criteria [34–37]. Fourteen more studies were excluded based on a review of the full text (see Fig. 1). Articles reporting on the same research project but describing different or new results were included as separate sources [22, 38], [39–41] and [42, 43]. A qualitative synthesis was conducted on a total of 24 studies.

### Quality assessment

The quality assessment of the included studies are presented in Additional file 2. For 19 of the studies that used a quantitative descriptive approach, it was unclear if the sample was representative of the population under study [21–24, 33, 38–42, 44–52]. Furthermore, 13 studies did not provide sufficient information regarding response rate [21, 23, 24, 33, 38, 40, 44, 47–52].

### Characteristics of included studies

Eight studies used a prospective observational design [22, 24, 38, 41, 44, 48, 49, 53], and five used a cross-sectional observational design [39, 40, 42, 43, 45]. Other studies used an action research design [52] or a pre- and post-test design [54, 55]. One was a pilot randomized controlled trial [56]. Seven studies did not report the study design [21, 23, 33, 46, 47, 50, 51] Fig. 1.

Participants were recruited using random sampling [51], purposive sampling [48], convenience sampling [39–41, 46, 47] and consecutive sampling [33]. In sixteen studies, the recruitment method was not reported [21–24, 38, 42–45, 49, 50, 52–56].

The characteristics of included studies are shown in Table 1. Twelve studies were conducted in Europe [21, 24, 33, 42–44, 47, 49, 52, 53, 55, 56], five in North America [39–41, 46, 51], one in Australia [48] and five in Asia [22, 23, 38, 45, 54]. One study [50] was a multinational collaboration between six countries. Of the 24 articles included, 23 were written in English, and one was written in Swedish [33].

**Table 1:** Characteristics of included studies

First author, year, country [reference]	Aim	Setting	Sample size *N* (n = number assessed with Doloplus-2)	Mean (SD)/Median (range) age, years	% women	Type of cognitive impairment and stage	Defined by authors as non-verbal/not able to self-report
Akbarzadeh, 2007, Sweden [33]	To psychometrically test the Swedish version of the Doloplus-2 instrument for its use among older people	Three acute and one psycho-geriatric ward in a hospital	*N* = 48	NR, sample > 65	NR	NR^†^	Yes
Ando, 2010, Japan [23]*	To develop a Japanese version of the Doloplus-2 and to apply it to elderly patients with Alzheimer’s disease (AD)	General hospital, surgical and psychiatric ward	*N* = 6	Mean 78.4 (4.7)	50%	Moderate to severe AD Mean MMSE 7.6 (5.5) and HDS-R 6.4 (5.6)	Yes
Ando, 2016, Japan [54]	To assess whether the Japanese Doloplus-2 scale could effectively identify pain in elderly individuals with moderate-to-severe dementia	Geriatric hospital	*N* = 39 (*n* = 19)	Mean 84.5 (6.6) experimental group Mean 87.5 (7.1) control group	79% (experimental group) 90% (control group)	Moderate to severe dementia Mean MMSE in experimental group 10.9 (SD 6.5), not reported for control group	The participants had to have the ability to say that they were currently in pain
Bauer, 2007, France [44]	To investigate the tolerability of equimolar mix in very elderly patients undergoing painful procedures	Hospital, geriatric short-stay unit	*N* = 62 (*n* = 43 procedures)	Mean 87 (5)	62.9%	54.8% of the participants were reported to have mild to moderate cognitive disorders	NR
Chen, 2010a, Taiwan [22]** ^a^	To translate the French version of the Doloplus-2 scale into Chinese and to evaluate the psychometric properties and the clinical feasibility of the translated instrument	LTC, five dementia special care units	*N* = 241	Mean 79.3 (9.4)	49%	Moderate to severe dementia Mean MMSE 5.26 (SD 5.46)	Yes
Chen, 2010b, Taiwan [38]^a^	To validate RNs’ and NAs’ report in assessing present pain and to investigate the potential influencing factors of institutionalized older people with dementia	LTC, six dementia special care units	*N* = 304	Mean 79.9 (8.8)	42%	Dementia 66% had a MMSE ≤10	The participant were asked about pain presences, intensity and location
Chen, 2014, Taiwan [45]	To test a causal model of the predictors of agitation	LTC, 11 dementia special care units	*N* = 405	Mean 80.6 (7.9)	33%	Varying stages of dementia 59.5% had a MMSE ≤10. Mean MMSE 8.9 (SD 6.8; range 0–26)	NR
Couilliot, 2013, France [55]	To investigate the acceptability and feasibility of an acupuncture intervention on persistent musculoskeletal pain in a geriatric population	Geriatric hospital	*N* = 60 (varying for the different time points n = 43 to *n* = 57)	Mean 83 (range 67–105)	80%	55.0% was diagnosed with dementia 62% were able to answer MMSE; mean 18.8 (SD 5.4)	63% of the participant diagnosed with dementia were able to self-rate their pain at the time of inclusion
Hadjistavropolous, 2008, Canada [46]	To examine the extent to which each of Doloplus-2’s items were predictive of delirium, depression, and dementia severity	Three LTC homes and a LTC unit within a large regional hospital	*N* = 160	Mean 86.3 (6.9)	70.6%	Varying stages of dementia Mean HDS 91.0 (SD 49.2)	NR
Hølen, 2005, Norway [21]	To translate the Doloplus-2 into Norwegian, to test the Doloplus-2 with regard to criterion validity and to obtain the administrators’ evaluation of the clinical performance of the Doloplus-2	NHs, three special units for dementia	*N* = 59	Median 82 (range 39)	80%	Dementia 88% had a MMSE <24; median 9 (Q1 = 3, Q3 = 18)	Yes
Hølen, 2007, Norway [47]	To test the criterion validity and inter-rater reliability of the Doloplus-2, and to explore a design for validations of behavioral pain assessment tools	Two NHs and a geriatric hospital unit	*N* = 41	Mean 84	74%	Cognitively impaired Median MMSE 10. 50% scored 0–10; 36% scored 11–20; 14% scored 21–30 on the MMSE.	Yes
Monacelli, 2013, Italy [53]	To re-assess pain after 1 year in a group of elderly NH residents with dementia	One NH	*N* = 23	Mean 88.1 (2.4)	78%	Moderate to severe dementia Mean MMSE 10.3 (SD 2.2)	Yes
Neville, 2014, Australia [48]	To evaluate the relative psychometric merits of the APS, the Doloplus-2 Scale, and the CNPI	Three residential aged care facilities	*N* = 126	Mean 85.2 (6.6; range 69–96)	83%	Moderate to severe dementia Mean GDS 5.7 (SD 1.5; range 0–7)	NR
Pautex, 2007, Switzerland [49]	To report the psychometric properties of the observational Doloplus-2 scale using the VAS pain score as a gold standard and evaluate its performance	A geriatric hospital and a department of psychiatry	*N* = 180	Mean 83.7 (6.5)	73%	74% had dementia (the remaining 26% had no cognitive decline). AD: 39%; Mixed: 34%; Vascular: 20%; Other causes: 5% Median MMSE 18.0 (±7.7). CDR score of 1 (mild dementia): 37%; score of 2 (moderate): 41%; score of 3 (severe): 33%	The participants had to have the ability to reliably use the VAS
Pickering, 2010, multinational [50]	To evaluate the translation of the Doloplus-2 scale in five languages, as regards test–retest and inter-rater reliability	Multicenter; NHs, LTC settings, rehabilitation, home dwelling, acute care, other	*N* = 341	Mean 82 ± 2	70%	Different incapacities; dementia, aphasia, behavioral disorders, ‘other’ MMSE range 0–12	Yes
Rodríguez-Mansilla, 2015, Spain [56]	To assess the effectiveness of ear acupressure and massage vs. control in the improvement of pain, anxiety and depression in persons diagnosed with dementia	Residential homes	*N* = 120	Range 67–91	77.4%	Dementia MMSE 0–20 was one of the inclusion criteria	NR
Sheu, 2011, Canada [51]	To examine the validity of facial expression components of 6 widely used pain assessment scales developed for elders with dementia	Hospital	*N* = 30	NR, sample > 65	NR	The majority had cognitive impairment	All patients had capacity to comprehend and communicate in English, so as to cooperate with instructions
Stacpoole, 2014, UK [52]	To evaluate the effects of the Namaste Care program on the behavioral symptoms of residents with advanced dementia in care homes and their pain management	Five dementia care homes	*N* = 37	Mean 78.5	59.4%	Severe dementia AD: 46%; Vascular: 19%; Mixed: 5.3%; Fronto-temporal: 2.7%; Unspecified: 27% BANS-S score ranged 17–28.	NR
Torvik, 2009, Norway [43]^b^	To describe the pain and use of pain medication in nursing home patients and examine which variables that were associated with pain	Seven NHs	Data abstracted is on the not self-reporting/proxy-rated group *N* = 86 (*n* = 77)	Mean 86 (6)	77%	MMSE was not scored because of cognitive impairment or lack of language	Yes
Torvik, 2010, Norway [42]^b^	To examine the use of Doloplus-2 in a nonverbal nursing home population, and to evaluate its reliability and validity by comparing registered nurses’ estimation of pain with Doloplus-2 scores	Seven NHs	*N* = 77	Mean 86 (6.6)	75%	None of the patients could complete the MMSE due to severe cognitive impairment, even though the majority had not been given any dementia diagnosis	Yes
Voyer, 2008, Canada [41]^c^	To determine detection rates of delirium and delirium symptoms by nurses among elderly residents with dementia and to identify factors associated with undetected cases of delirium	Three LTC facilities and one LTC unit of a large hospital	*N* = 156 (*n* = 109)	Mean 86.3 (6.9)	73.7%	Dementia Early: 3.1%; Middle:; 72.1%; Late: 24.8% according to FAST score AD: 34.2%; Vascular: 18.1%; Mixed: 14.2%; Subcortical: 4.5%; Unspecified: 29.1%	NR
Voyer, 2009, Canada [39]^c^	To investigate predisposing factors associated with delirium among demented long-term-care residents and to assess the cumulative effect of these factors on the likelihood of having delirium	Three LTC facilities and one LTC unit of a large regional hospital	*N* = 155	Mean 86.3 (6.9)	73.6%	Dementia Mean HDS 91.1 (SD 48.9)	NR
Voyer, 2011, Canada [40]^c^	To investigate individual and environmental factors associated with delirium severity among older persons with delirium superimposed on dementia	Three LTC facilities and one LTC unit of a large regional hospital	*N* = 71	Mean 87.7 (7.4)	71.8%	Dementia Mean HDS 79.6 (SD 43.5)	NR
Zwakhalen, 2006, the Netherlands [24]	To evaluate the psychometric properties of translated versions of the PAINAD, PACSLAC, and Doloplus-2 scales	NHs, 12 psycho-geriatric wards and a somatic NH ward	*N* = 128 (assessed at rest T1 *n* = 89, at specific moment T3 *n* = 26)	Mean 82.4 (6.8)	78%	Dementia AD: 32.0%; Vascular: 18.8%; Other (e.g. Parkinson’s disease, frontal lobe): 5.5%; Mixed: 3.9%; Unknown: 8% Mild: 21.9%; Moderate to moderately severe: 28.1%; Severe to very severe: 47.7%; Unknown/missing: 2.3% according to CPS score	Patients were questioned about their current pain intensity using self-report scales

AD: Alzheimer’s Disease; APS: Abbey Pain Scale; BANS-S: Bedford Alzheimer’s Nursing Severity Scale; CDR: Clinical Dementia Rating scale; CNPI: Checklist of Nonverbal Pain Indicators Scale; CPS = Cognitive Performance Scale; FAST = Functional Assessment Staging; GDS: Global Deterioration Scale; HDS = Hierarchic Dementia Scale; HDS-R: Hasegawa Dementia Scale – Revised; LTC = Long-term Care; RN = Registered Nurse; NA = Nursing Assistant; NH = Nursing home; NR: Not reported; MMSE: Mini-Mental State Examination; Pain Assessment Checklist for Seniors with Limited Ability to Communicate; PAINAD: Pain Assessment in Advanced Dementia; SD: Standard Deviation; VAS: Visual Analogue Scale

^†^e-mail correspondence with corresponding author: all participants were older adults with communication difficulties, some, but not all had cognitive impairment

^*^This study report on several phases (Translation; Implementation of Version 1 and 2; Nurses’ experience implementing Version 1 and 2). Data abstraction is solely on Version 2

^**^This study report on three phases (I: Translation; II: Pilot testing; III: Validation of the psychometric properties of C-Doloplus-2). Data abstraction is solely on phase III

^a,b,c^Articles reporting on the same study

For studies reporting on inter-rater reliability, we considered the patients to be the sample, not the assessors

Eleven studies were conducted in a LTC setting [21, 22, 24, 38, 42, 43, 45, 48, 52, 53, 56]. Others were conducted in a hospital [23, 33, 44, 49, 51, 54, 55] or a combination of various settings [39–41, 46, 47, 50]. The sample sizes ranged from *N* = 6 [23] to *N* = 405 [45] participants; the percentage of female participants ranged from 33% [45] to 83% [48]. The mean age of participants ranged from 78.4 [23] to 88.1 [53]. Four studies [44, 49, 51, 55] used mixed samples that included patients with and without cognitive impairment. The ability of the participants to self-report pain varied across the included studies; nine defined the participants as nonverbal or unable to self-report pain [21–23, 33, 42, 43, 47, 50, 53], while in other studies, all of the participants were able to self-report their pain [24, 38, 49, 51, 54, 55]. For nine studies, the authors did not report the participants’ abilities to self-report pain or communicate verbally [39–41, 44–46, 48, 52, 56].

### Feasibility and clinical utility

Table 2 shows the studies that examine the feasibility and the clinical utility of the Doloplus-2. Only four studies explicitly address feasibility and/or clinical utility [21, 22, 24, 53], but relevant information was also found in other studies. The mean totals of the Doloplus-2 baseline measurement ranged from 3.5 [45] to 22.7 [56]. All but two studies used all ten items in the scale [44, 51]. Every study that applied a cut-off used the recommended cut-off of ≥5 out of 30 [21–24, 33, 38–43, 45, 48, 49, 52–54, 56]. The percentage of participants who scored above the cut-off (indicating pain) ranged from 19% [49] to 96% [53].

**Table 2:** Feasibility and clinical utility of the Doloplus-2

First author, year, country [reference]	Assessment completed by	Cut-off, % who scored ≥ cut-off	Number of items used	Mean (SD) /Median (range) score, total and subscales	Time needed to complete the assessment	Training given on Doloplus-2	Raters’ knowledge of the patients’ normal behavior	Other information about feasibility and/or clinical utility
Akbarzadeh, 2007, Sweden [33]	RNs and NAs	≥5^*^	10	Mean total score 11.9 (6.5) for rater 1 and 12.6 (6.8) for rater 2 Median total score 12 (0–25) for rater 1 and rater 2	NR	NR	NR	NR
Ando, 2010, Japan [23]	First author and authors (CA) and RNs	≥5^*^	10	NR	NR	All nurses were provided with in-depth instructions regarding scoring of the Doloplus-2	At admission to hospital, nurses observed the patients’ behavior in an attempt to learn their habits and usual condition by talking with family or health care workers who were familiar with the patient	Nurses’ (*N* = 14) were interviewed and the scale was said to be feasible
Ando, 2016, Japan [54]	RNs	≥5, 79%	10	Mean total score Pre-test 7.5 (3.2) Post-test 2.9 (2.1)	NR	One of the authors held meetings with the RNs to provide in-depth instructions regarding scoring of the Doloplus-2	NR	The experimental group, who was assessed with the Doloplus-2, received pain medication significantly more frequently than the control group who was not assessed with Doloplus-2: χ2 [1, 40] = 16.0, *p* < 0.001; φ = 0.6, and the mean pain score significantly decreased post treatment (*p* < 0.001)
Bauer, 2007, France [44]	NR	NR	5; Somatic complaints, Protection of sore areas, Expression, Communication and Problems of behavior	NR	NR	NR	NR	NR
Chen, 2010a, Taiwan [22]	RNs and RAs	≥5, 39.8%	10	Mean score Total 4.5 (4.1); Somatic 1.3 (2.1); Psychomotor 2.1 (1.9); Psychosocial 1.1 (1.9)	NR	RNs in each institution received intensive training from the researcher in the use of the C-Doloplus-2, following the user manual	RNs must have worked in their dementia special care unit at least one month before data collection began To ensure RAs familiarity with residents, they were asked to observe and record resident’s painful conditions at rest and after pain-provoked motion every day for one week	Nurses (*N* = 14) asked to rate on a 5-point Likert-type scale (5 = strongly agree to 1 = strongly disagree) “Do you think the C-Doloplus-2 is appropriate for assessing pain in cognitively impaired older people with communication difficulty?” Mean score 4.1 (SD 0.8; range 3–5) The RNs indicated it was difficult to distinguish whether there are behavioral changes in sleep pattern, communication and social life of older people with end-stage of dementia, but most agreed that the C-Doloplus-2 scale has clinical potential to detect pain in this group
Chen, 2010b, Taiwan [38]	RNs and NAs from the units and RAs with a Bachelor of Science or higher degrees and majors in psychology or nursing	≥5, 34% (RNs); 48% (NAs); 38% (RAs)	10	NR	NR	RAs underwent a series of training courses; five hours of instruction about pain in older people with dementia and two weeks of clinical practice training about performing self-report and observational instruments to assess pain in older people with dementia	RNs and NAs must have worked in their dementia special care unit at least one month before data collection began RAs observed resident’s painful conditions at rest and after pain-provoked motion and interviewed residents about their pain every day for one week prior to assessment with Doloplus-2	NR
Chen, 2014, Taiwan [45]	RAs with Bachelor of Science or higher degrees and majors in psychology or nursing	≥5, 33.8%	10	Mean total score 3.5 (3.2; range 0–15)	NR	The RAs received 6 h of instruction pertaining to pain, depression and agitation in dementia, and two weeks of skills training in observing and recording in clinical settings	For one week, the RAs observed the residents’ behavior directly as they performed ADL, noting pain behaviors	NR
Couilliot, 2013, France [55]	The hospital’s caregivers	NR	10	Baseline mean scores Total 8.7 (4.7); Somatic 4.7 (2.6); Psychomotor 2.3 (1.3); Psychosocial 1.6 (2.2)	NR	The hospital’s caregivers had been previously trained and were competent in assessment with the scale	NR	NR
Hadjistavropolous, 2008, Canada [46]	Research nurses	NR	10	Mean total score 4.5 (4.4)	NR	Research nurses completed 15 h of instruction on delirium, dementia, and depression from a member of the research team. Instruction on the research procedures as well as direct supervision in the data collection for 15 participants were also provided	NR	NR
Hølen, 2005, Norway [21]	Nurse in co-operation with a RA	≥5, 49%	10	Mean total score 5.2 (5.2)	NR	Nurses and RAs trained in accordance with the Doloplus-2 standard recommendations	The nurses administering the scale worked close to the patients and were familiar with their habits and regular condition	A debriefing questionnaire was completed by the administrators of the Doloplus-2 (*N* = 11): - The standardized format makes the discussion of a patient more solid - Small enough administrative burden and usable in routine care situations - Items eight to ten (psychosocial reactions), should be cautiously scored because changes in these behavior can be a result of dementia, and not necessarily pain. Therefore, it is important to know the patient’s habits and regular behavioral patterns - Training and reading of the instruction manual are important for using the scale correctly
Hølen, 2007, Norway [47]	RNs	5 out of 30^*^	10	Mean total score 7.5 (5.1; range 0–22)	NR	Nurses who used the Dolplus-2 were trained, but no details provided	RNs administering the scale cared for the patients regularly and were familiar with their behavior	NR
Monacelli, 2013, Italy [53]	A nurse working in the NH	Higher than 5/30, 96%	10	NR	Average 8–10 min per patient	Adequate professional training with reference to Pickering, 2010 [54]	NR	Collection of professional comments on the administration of the scale defined it as handy and easy for clinical application and mostly suitable for a residential setting were professionals are engaged with a daily care of patients After assessing with Doloplus-2 for 1 year: - Reduced mean score below the pain threshold: Chi square = 14.8; *p* < 0.0001. - Increased analgesic therapy: At the initial assessment, analgesic therapy was of 30% with only 1 level WHO group. After 1 year, the analgesic treatment was of 100% with 1 level WHO group of 15%, 2 level WHO group of 75% and 3 level WHO group of 10%
Neville, 2014, Australia [48]	RNs, enrolled nurses and assistants-in-nursing	5^*^	10	Mean total score First testing occasion: 9.0 (6.5) for rater 1 and 7.4 (6.2) for rater 2. Second testing occasion: 7.1 (6.0) for rater 1 and 6.8 (5.9) for rater 2	NR	The nurses as rater of the Doloplus-2 scale, received training from a project team member, but no more details provided	The nurses were well aware of the person they were assessing	Nurse qualification was significantly associated with Doloplus-2 score at the first testing occasions (R^2^ = 0.1; *p* = .004). More highly qualified nurse raters tended to assign higher pain ratings. The scale may initially be susceptible to rater qualification, but this effect disappears with repeated use There was no significant effect from different nurse raters producing pain ratings, over and above the effects of rater demographics (all *p* > 0.12), indicating that multiple raters does not bias pain scores
Pautex, 2007, Switzerland [49]	Nurses	≥5, 19%	10	Median total scale 4 (interquartile range 7)	Average 10 (6 to 12) minutes per patient	A nurse at each unit received extensive training to complete Doloplus-2 and had the responsibility to train other nurses in the unit for at least 1 h and supervised their use of the scale	NR	Constructed and tested a shortened version of the Doloplus-2 (5 items). Internal consistency and correlation with VAS was similar to the complete Doloplus-2 Of the 88 patients who reported pain using VAS, 50 got a score lower than 5 and 21 got a score equal to 0 on the Dolplus-2. Patients report more pain using self-report (VAS) than nurses uncover with the Doloplus-2
Pickering, 2010, multinational [50]	Two physicians per team (9 teams)	NR	10	Mean total sore per language version - Dutch: 5.4 (4.4) for rater 1 and 4.1 (3.8) for rater 2 - English: 8.3 (6.0) for rater 1 and 8.8 (6.5) for rater 2 - Italian: 12.7 (6.5) for rater 1 and 12.7 (6.8) for rater 2 - Portuguese: 6.1 (7.0) for rater 1 and 6.2 (7.0) for rater 2 - Spanish: 6.0 (4.9) for rater 1 and 6.3 (4.6) for rater 2	Average 5 min per patient	The team was provided with Doloplus-2 video, instructions for use, several evaluations with paper and video backups. Implemented the scale a few days before study start to familiarize themselves with it	All physicians were familiar with the patient and provided daily medical care	All participating physicians considered Doloplus-2 to be easy to use once they were familiar with it
Rodríguez-Mansilla, 2015, Spain [56]	An occupational therapist	Scores over 5^*^	10	Mean total score baseline: Ear acupressure 19.0 (5.1); Massage 22.7 (6.4); Control: 21.4 (2.7)	NR	NR	NR	NR
Sheu, 2011, Canada [51]	“Coders” over 19 years of age with healthy vision was recruited from a university campus	NR	1, only the ‘Facial expression’ item	NR	NR	NR	NR	NR
Stacpoole, 2014, UK [52]	Researcher with care staff	5 or more^*^	10	NR	NR	NR	NR	NR
Torvik, 2009, Norway [43]	RNs	5, 67.5%	10	NR	NR	The researcher trained the RNs in data collection and was available during data collection	The RNs were the patients’ primary nurses who cared for the patient regularly	NR
Torvik, 2010, Norway [42]	RNs	5, 68%	10	Mean score Total 6.9 (4.4); Somatic 3.5 (2.7); Psychomotor 1.6 (1.3); Psychosocial 2.0 (2.4)	NR	The researcher increased staff awareness of patients’ pain by teaching about pain and Doloplus-2. Staff received both oral and written information about how to use the Doloplus-2	The RNs were the patients’ primary nurses who cared for the patient regularly	The highest congruency between Doloplus-2 score > 5 and RNs reporting ‘Don’t know’ when proxy-rating pain, was found on the Psychosocial subscale The highest congruence between the Doloplus-2 score and the proxy-rating occurred on the Psychomotor score RNs evaluated significantly more patients as experiencing pain compared with proxy-rated pain (*p* = 0.001)
Voyer, 2008, Canada [41]	RAs who were nurses	5 out of 30, 44%	10	NR	NR	NR	NR	NR
Voyer, 2009, Canada [39]	Study nurses	5 out of 30, 45.8%	10	NR	NR	NR	NR	NR
Voyer, 2011, Canada [40]	Study nurses	5 out of 30, 50.7%	10	NR	NR	NR	NR	NR
Zwakhalen, 2006, the Netherlands [24]	Nurses	5 out of 30^*^	10	Mean total score ‘Daily pain’ group 9.8 (6.0; range 2–23). ‘No pain group’ 5.1 (3.9; range 0–16)	NR	NR	P.212: “…the Doloplus-2 cannot be used without in-depth knowledge of the patient…”, “but not specify raters’ knowledge of the patients’ normal behavior”	Nurses’ (*N* = 12) ratings of clinical usefulness (scored on a 10-point scale): mean 5.6 (SD 2.2) Qualitative information from nurses, p.: 217: “Doloplus-2 provides a more general view. A clear manual is provided. The scale is difficult to score and interpret. It’s questionable whether all items of the Doloplus are relevant to detect pain. The psychosocial items in particular are difficult to interpret as solid specific pain behavior. Other causes, like the dementia itself, could explain a change in psychosocial behavior.”

NR: Not reported; NA: Nursing Assistant; NH: Nursing Home; RA: Research Assistant; RN: Registered Nurse

*Only referring to the Doloplus-2 home page or articles published by the Doloplus-group, do not apply the cut-off in their study

#### Feasibility

Fifteen studies reported, in varying detail, that the raters received some form of training in how to use the Doloplus-2 to collect data [21–23, 38, 42, 43, 45–50, 53–55]. Nine of the studies included clear (but brief) information about the content of the training [21–23, 38, 42, 45, 46, 50, 54]. The training method was reported in nine studies [22, 38, 42, 43, 45, 46, 49, 50, 54], and six reported the duration or amount of training [7, 22, 38, 45, 46, 49]. Every study that described the trainer reported that a member of the research team provided the training [22, 39–43, 46, 48, 49, 54]. Two studies simply mention that training was provided without providing any details [47, 55], and one [53] refers to the procedure of another study [50]. In one study, raters gave feedback on the importance of being trained in data collection using the Doloplus-2 and of knowing the patients’ normal behaviour in order to use the Doloplus-2 correctly [21].

Ten studies specified that the raters were familiar with the patients’ normal behaviour [21–23, 38, 42, 43, 45, 47, 48, 50]. In the remaining studies, this was not clear or not reported. Most of the Doloplus-2 assessments were conducted by a person with a background in nursing [21–24, 33, 38–43, 45–49, 53, 54], sometimes in collaboration with research assistants (RA) or a researcher. In other studies, physicians [50] or an occupational therapist [56] performed the assessments. A description of the raters was not provided or was unclear in four studies [44, 51, 52, 55]. One study reported the initial impact of nurses’ qualifications: More highly qualified nurse raters tended to assign higher pain ratings on the Dololpus-2. The effect of nurse qualifications seemed to disappear with repeated use of the scale, and the number of raters did not bias the result [48].

On average, it took raters five to ten minutes per patient to complete the Doloplus-2 [49, 50, 53]. The raters thought that the scale’s administrative burden was small [21]. They also thought that the Doloplus-2 was feasible [23] and easy to use [50, 53] and that the manual was clear [24].

#### Clinical utility

In one study, after a year of regular Doloplus-2 assessments, patients’ pain scores decreased significantly, and HCPs’ use of analgesic therapy with non-opioids (Step 1 of the WHO pain ladder) increased significantly, from a baseline of 30% to 100% [53]. In another pre- and post-test study, participants in the experimental group were assessed with the Doloplus-2 and received significantly more analgesics than the control group, which was not assessed with the Doloplus-2 [54].

Some studies also evaluated the Doloplus-2’s usefulness. One study found that the scale was useful in assessing pain [22], whereas another study reported that the Doloplus-2 was the least useful of the three pain scales evaluated [24]. The scale has been reported to facilitate valuable discussions about patients [21]. Raters using the Doloplus-2 stated that the psychosocial items were difficult to understand and score [22, 24] and that these items should be cautiously scored because abnormal social reactions can also be caused by dementia [21]. Furthermore, the highest congruency between Doloplus-2 scores over 5 and registered nurses (RN) reporting ‘don’t know’ when proxy-rating pain was found on the psychosocial subscale [42].

When comparing the Doloplus-2 with other methods used to assess pain in older adults with cognitive impairment, one study in a nursing home found that nurses evaluated significantly more patients as having pain when using Doloplus-2 than when proxy-rating pain. With proxy-rating alone, nurses were not able to say whether one-third of the patients appeared to be in pain [42]. A second study found that patients reported more pain using the Visual Analogue Scale (VAS) than nurses did using the Dolplus-2 [49]. The same study also found that of all the patients who self-reported pain, only one in five scored ≥5 on the Doloplus-2. This raises the question of whether the cut-off score should be adjusted [42, 49]. The different study populations (verbal and nonverbal) may explain the different results. It is possible that pain behaviour in people who are able to self-report is different to that of people who cannot self-report due to more advanced cognitive impairment.

### Measurement properties

Seventeen studies reported on one or more measurement properties of the Doloplus-2 (Table 3).

**Table 3:** Measurement properties of the Doloplus-2

First author, year, country [reference]	Reliability	Validity	Responsiveness	Interpretability
Akbarzadeh, 2007, Sweden [33]	Internal consistency Cronbach’s alpha for the total scale 0.84 for rater 1 and 0.82 for rater 2 Reliability (Inter-rater) Agreement between rater 1 and rater 2 for single items (Cohen’s Kappa coefficient) 0.31–0.69 No statistically significant difference between rater 1 and rater 2 for total score (Wilcoxon signed-rank test) *p* = 0.106 Spearman correlation 0.90 between rater 1 and rater 2 for total score	Criterion (Concurrent) Spearman correlation with the UAB as the ‘gold standard’ 0.70 for rater 1 and 0.72 for rater 2 Construct (Structural) EFA with the result of items loading on one factor	NR	NR
Ando, 2010, Japan [23]	Reliability (Inter-rater) Matching scores by RN and researcher 77.5%, *p* = <0.01 The ICC for the agreement between RN and researcher was 0.90 (*p* = 0.001). Agreement by items 0.67–0.96	Construct (cross-cultural) Semi-structured interviews with 14 nurses. Two items, ‘Protective body postures adopted at rest’ and ‘Sleep pattern’, were changed to more appropriate Japanese explanations	NR	NR
Ando, 2016, Japan [54]	NR	NR	Before treatment, the mean total score was 9.8 (SD 4.2) for *n* = 10 patients, whereas their post-treatment score significantly decreased to 2.7 (SD 1.6); net change 7.1, 95% CI: 4.4–9.7	NR
Bauer, 2007, France [44]	NR	NR	NR	NR
Chen, 2010a, Taiwan [22]	Internal consistency Cronbach’s alpha for the total scale 0.74. Subscales Somatic 0.79; Psychomotor 0.87; Psychosocial 0.74. The alpha coefficients did not increase when any of the items were deleted Reliability (Inter-rater) ICC for the agreement between RNs and RAs on the total scale 0.81. For the subscales; Somatic 0.79, Psychomotor 0.84 and Psychosocial 0.60	Construct (Hypotheses testing) Pearson correlations with known correlates of pain. In moderate dementia, significant correlation with functional ability −0.38 (*p* < 0.01). In severe dementia, significant correlation with functional ability −0.22 (p < 0.01) and depression 0.12 (*p* < 0.05) Construct (Structural) A PCA showed three factors, accounting for 65% of the total variance. Factor 1: all five items of the Somatic subscale explained 27.43% of the variance. Factor 2: all three items of the Psychosocial subscale explained 19.86% of the variance. Factor 3: both items of the Psychomotor subscale, accounting for 19.99% of the variance Item-total and item-subtotal correlations: Each item was correlated with the originally belonged subscale, ranged from 0.6 to 0.94. Each item correlation with overall scale ranged from 0.42 to 0.65 Construct (Cross-cultural) Five experts examined the content of C-Doloplus-2 and rated each item on a 4-point Likert scale from relevant (4) to irrelevant (1). Only the option ‘Insomnia, affecting morning waking time’ of item 5 ‘Sleep pattern’ was recommended to be rephrased	NR	NR
Chen, 2010b, Taiwan [38]	Reliability (inter-rater) Paired t-test for agreement of different pairs in assessing pain. No difference between mean total scores for RA-RN pairs (*t* = 0.28, *p* > 0.05), but a statistically significant differences between the mean total scores for RA-NA pairs (*t* = 6.70, p < 0.01). NAs tended to report more pain cues than RAs Logistic regression to examine factors influencing the extent of agreement for the different pair. For RA-RN pairs, OR increased when residents had stayed in the institution longer (OR 1.01, *p* = 0.01), had less physical dependency (OR 1.02, p = 0.00), and when RNs had received pain related training (OR 2.86, *p* = 0.04). For RA-NA pairs, OR increased when the patients had fewer medical diagnosis (OR 0.78, p = 0.01) and less physical dependency (OR 1.01, p = 0.04)	NR	NR	NR
Chen, 2014, Taiwan [45]	Internal consistency Cronbach’s alpha for total scale 0.73	NR	NR	NR
Couilliot, 2013, France [55]	NR	NR	Statistically significant reduction on total and subscales scores after five acupuncture sessions: Total score mean variation −3.27, *p* < 0.01, effect size 0.77. Somatic score mean variation −2.08, *p* < 0.01, effect size 0.89. Psychomotor score mean variation −0.61, *p* < 0.05, effect size 0.33 Psychosocial score mean variation −0.59 points, *p* < 0.05, effect size 0.30	NR
Hadjistavropolous, 2008, Canada [46]	NR	Construct (Hypotheses testing) Item 10 (‘Problems of Behavior’) was related to dementia severity (β = − .25, *p* < .003), depression (β = .31, *p* < .001) and presence of delirium (β = .25, *p* < .003) Item 6 (‘Washing and dressing’) was related to delirium severity (β = .42, *p* < .004) and dementia severity (β = − .39, *p* < .005) Other items related to depression were item 5 (‘Sleep pattern’) (β = .22, p < .003), item 9 ‘Social life’ (β = .25, *p* < .001)	NR	NR
Hølen, 2005, Norway [21]	NR	Content (Face) Results from a questionnaire, completed by the 11 administrators of the Doloplus-2, was the Doloplus-2 was instructive regarding observation indicating pain, and includes important pain clues Construct (cross-cultural) The translation was approved by all administrators. No item was pointed out as confusing, difficult to understand or elsewhere problematic Criterion (concurrent) Experts’ pain rating with NRS-11 was used as a pain criterion. The experts rated 25 patients as pain free where the Doloplus-2 made five false positive with scores of 5 and 6. Of the 59 cases, the Doloplus-2 made false negatives on 10 occasions: a Doloplus-2 ≥ 5 at the same time as the expert rated above 0 on the NRS-11. In five of these cases, the expert’s score was one half (usually 0 at rest and 1 in movement), three had a score of 1 and the remaining two were rated with 2 and 3 on the NRS-11 The Doloplus-2 explained 62% (R^2^) of the pain distribution. For 85% of the assessments, the Doloplus-2 score (0–30) multiplied by 0.25 (beta) corresponded to the expert score ± 1 unit on the 0–10 NRS scale Facial expression explained 48% (R^2^ = 0.48) of the experts scores alone. When including items Protective body postures at rest, Communication and Somatic complaints, these four items explained 68% of the total variability in the experts’ scores	NR	NR
Hølen, 2007, Norway [47]	Reliability (inter-rater) Agreement between a geriatric specialist nurse and an enrolled nurse on the total score was 0.77 (ICC), with a 95% CI of 0.47–0.92. Assessed in the 16 patients included at the geriatric hospital unit	Criterion (concurrent) The pain criterion was the specialist nurse (pain expert) who made a single evaluation of each patient’s pain level on NRS-11. Doloplus-2 scores against the expert scores produced an R^2^ = 0.023, implying poor criterion validity of the Doloplus-2 when compared to pain experts evaluation. Association was found between the pain expert and the geriatric expert nurse who administered the Doloplus-2 in 16 patients in the Hospital, R^2^ = 0.54	NR	NR
Monacelli, 2013, Italy [53]	NR	NR	Reduction of total mean score between the first assessment and after 1 year of follow up (Wilcoxon rank test) R^2^ = 0.216, *p* < 0.001	NR
Neville, 2014, Australia [48]	Internal consistency Cronbach’s alpha for the two rater groups on the two assessment occasion was 0.86 and 0.87 Reliability (test–retest) Agreement for the two testing occasions occurring two weeks apart. Pearson correlation 0.71 for both rater groups Reliability (inter-rater) ICC for the agreement between nurse raters for the total score at first 0.73 and second testing occasion 0.81. Weighted Kappa to compare pain level categorizations (no pain, mild, moderate, severe pain) across raters at first 0.42 and second testing occasion 0.50	Criterion (concurrent) Pain criterion was RNs initial yes/no rating of the residents’ pain. Pearson correlation for each rater group at the first testing occasion showed moderate correlations at 0.43 (rater group 1) and 0.45 (rater group 2) Construct (Structural) EFA showed a 1-factor solution was the best description of the factor structure of the Doloplus-2 EFA showed a single factor model best described the correlation among all the total scale scores for the Doloplus-2, CNPI and APS, each score loading highly (>0.60) on that single factor, indicating that all of the scales measures essentially the same single construct		
Pautex, 2007, Switzerland [49]	Internal consistency Cronbach’s alpha was adequate for all items, lower in patients with dementia (0.67) compared to cognitively intact patients (0.84). The lowest internal consistency scores were found for the items ‘Expression’ (0.82) and ‘Mobility’ (0.82) Reliability (test-retest) Performed in a subsample of 20 patients hospitalized in the same units with the same characteristics and stable chronic pain. The second testing occasion happened the day after the first one. ICC indicated excellent agreement at 0.96	Criterion (concurrent) Spearman 0.46 indicated a moderate correlation with the pain criterion; patients’ self-assessment (VAS). The correlation was better in patients without dementia compared to patients with dementia (0.68 vs. 0.38) Doloplus-2 predicted 41% of the variability of pain intensity measured by VAS. The somatic dimension explained 36% of the variability, the psychomotor and psychosocial dimension 5% each. The intensity of pain (VAS) was mainly associated with the somatic dimension of Doloplus-2. Two items of the psychosocial reaction were also statistically significant (*p* < 0.05)	NR	NR
Pickering, 2010, multinational [50]	Reliability (test-retest) Patients was assess at initial contact and again 4 h later. When evaluated with ICC, agreement ranged from 0.62 for the Dutch version to 0.98 for the Italian version (0.98). Evaluated with Pearson correlation, the results ranged from 0.57 for the Dutch version to 0.99 for the Portuguese version Reliability (inter-rater) ICC for the agreement between physicians for the total score ranged from 0.75 (Dutch version) to 0.97 (Italian version) Pearson correlation indicated excellent agreement ranging from 0.75 (Dutch version) to 0.97 (Italian and Portuguese version) Kappa was used to compare agreement for each of the 10 items across language version. The agreement ranged from fair to excellent (0.51–0.84) for the English version, excellent (0.79–0.96) for the Italian version, good to excellent (0.65–0.82) for the Portuguese version, fair to excellent (0.47–0.87) for the Spanish version and poor to excellent (0.19–1) for the Dutch version	NR	NR	NR
Rodríguez-Mansilla, 2015, Spain [56]	NR	NR	The best improvement in the mean total score was reached in the last (third) month of ear acupressure. The average improvement was 8.55 points (SD 4.39), 95% CI: 7.14–9.95	NR
Sheu, 2011, Canada [51]	Reliability (inter-rater) Three clips indicative of mild, moderate and severe pain intensities were selected for study for each participant. The mean of criterionvalues for each intensity level was 0.04 (−0.20–0.38) for mild pain, 0.20 (−0.07–0.46), for moderate pain, and 0.38 (0.11–0 .68) for severe pain	Criterion (concurrent) Pain criterion used was FACS-scores. No significant correlations were observed with the FACS at any of the pain intensities. Pearson correlation for mild pain was −0.13 (an inverse relationship between scores), 0.16 for moderate pain, and 0.10 for severe pain Construct (Hypotheses testing) Examined whether the scale differentiated the 3 levels of facial expression of pain by a pairwise comparison of the mean between each intensity level of the scale. The Doloplus-2 did not distinguish the 3 levels of pain: Mild-moderate: 0.37, *p* = 0.488 Mild-severe: 0.03, *p* = 0.955 Moderate-severe: 0.40, *p* = 0.481	NR	NR
Stacpoole, 2014, UK [52]	NR	NR	NR	NR
Torvik, 2009, Norway [43]	NR	NR	NR	NR
Torvik, 2010, Norway [42]	Internal consistency Cronbach’s alpha for the total scale was 0.71, and 0.60 (Somatic) 0.80 (Psychomotor) and 0.78 (Psychosocial) for the subscales. After excluding individual items, the alpha values for the subscales were comparable to alpha for the overall scale, except for the Somatic subscale where the alpha score decreased from 0.60 to 0.47 when item ‘Somatic complaint’ deleted	Criterion (concurrent) Pain criterion used was RNs proxy assessment answering the question ‘Do you believe that this patient is experiencing pain?’ Response options were ‘no’, ‘yes’ or ‘don’t know’. Nursing staff evaluated significantly more patients as experiencing pain when using Doloplus-2 compared with proxy-rated pain (*p* = 0.01) When pain was proxy rated, 36 of 40 (90%) cases where the RNs assessed ‘yes, pain’, scored ≥5 on Doloplus-2. 11 of 15 (73.3%) assessed as ‘no pain’ by RNs scored <5 on Doloplus-2	NR	NR
Voyer, 2008, Canada [41]	NR	NR	NR	NR
Voyer, 2009, Canada [39]	NR	NR	NR	NR
Voyer, 2011, Canada [40]	NR	NR	NR	NR
Zwakhalen, 2006, the Netherlands [24]	Internal consistency Internal consistency for the total and subscales at different assessment points (T1 and T3). Cronbach’s alpha was 0.75 for the total scale, 0.70 for Somatic reactions, 0.80 for Psychomotor reactions, and 0.63 for Psychosocial reactions at T1 At T3, Cronbach’s alpha was 0.74 for the total scale, 0.63 for Somatic reactions, 0.77 for Psychomotor reactions, and 0.58 for Psychosocial reactions	Construct (Hypotheses testing) Used the known-groups technique by comparing Doloplus-2 scores between a ‘non-pain’ group’ and a ‘daily pain’ group. The mean score in the ‘Daily pain’ (mean 9.8; SD 6.0; range 2–23) was obviously higher compared to mean score in ‘no pain group’ (mean 5.1; SD 3.9; range 0–16) Pearson correlation was 0.29 for VAS by rater 1, 0.33 for VAS by nurse, 0.36 for the VRS, 0.29 for the PACSLAC and 0.34 for the PAINAD		NR

APS: Abbey Pain Scale; CI: Confidence Interval; CNPI: Checklist for Nonverbal Pain Indicators; EFA: Exploratory factor analysis; FACS: Facial Action Coding System; ICC: Intra-Class Correlation; NA: Nursing Assistant; NR: Not reported; NRS: Numerical Rating Scale; OR: Odds ratio; PACSLAC: Pain Assessment Checklist for Seniors with Limited Ability to Communicate; PAINAD: Pain Assessment in Advanced Dementia; PCA: Principal Component Analysis; RN: Registered Nurse; RA: Research Assistant; SD: Standard deviation; UAB: University Alabama Birmingham Pain Behavior Scale; VAS; Visual Analogue Scale; VRS: Verbal Rating Scale

#### Reliability

##### Internal consistency

The Cronbach’s alpha for the total scale ranged from 0.67 [49] to 0.84 [33, 49], indicating low to moderately good internal consistency across settings. The alpha coefficients for the total scale did not increase when any of the items were deleted [22], but they were lower for patients with dementia than for those who were not cognitively impaired [49]. The items in the Doloplus-2 are heterogeneous, so they are not expected to correlate well with each other since they reflect a variety of dimensions [42].

The Cronbach’s alpha for the subscales ranged from low to moderate or good internal consistency in the different settings, including nursing homes (0.60 to 0.84) [22, 42].

##### Test-retest reliability

Test-retest reliability was high to excellent in one study in a hospital setting (Intraclass Correlation Coefficient (ICC) = 0.96) [49]. The test-retest reliability for multilingual versions of the test in multiple settings was moderately good to high or excellent; the ICC ranged from 0.62 (the Dutch version) to 0.98 (the Italian version) [50].

##### Inter-rater reliability

Inter-rater reliability was tested using different statistical techniques (ICC, Pearson correlation, Kappa statistics, Wilcoxon signed rank, paired t-test, matching scores) [22, 23, 47, 48, 50]. Agreement among raters ranged from 0.73 [48] to 0.97 [50], indicating moderately good to high or excellent inter-rater reliability across settings. Agreement for the subscales ranged from 0.60 to 0.84 [22]. One study compared pain level categorizations (the Doloplus-2 total score was used to classify patients into groups with mild, moderate or severe pain) across raters and found moderately good agreement (0.42 and 0.50) on two testing occasions [48]. The mean κ values for pairs of raters at each pain intensity level (mild, moderate, severe) increased as pain intensity increased (from mild 0.04 to severe 0.38) [51]. High intensity behaviour is more obvious and most likely easier for raters to spot and agree on. One study found no statistically significant differences between the two raters in the total score [33]. Another study found no difference between mean total scores for RA-RN pairs but found a statistically significant difference between the mean total scores of RA-Nursing Assistant (NA) pairs; the NAs reported more pain cues than the RAs [38]. In another study, matching scores by researchers and RNs was 77.5%, p = <0.01 [23].

#### Validity

##### Content validity

The degree to which the (items of an) instrument seems to be an adequate reflection of the construct to be measured was only addressed in one study, which reported that that the scale pinpoints important pain clues [21].

##### Construct validity

A 1-factor solution was the best description in two studies using exploratory factor analysis [33, 48]. In a study using principal component analysis, items loaded on three factors, and each item was correlated with the originally belonged subscale in addition to the overall scale [22]. A single-factor model best described the correlation between Doloplus-2 and two other observational pain assessment tools (the Abbey Pain Scale and the Checklist of Nonverbal Pain Indicators), indicating that these scales measure the same single construct [48].

Cross-cultural validity was examined in three studies. In these, a group of experts or the raters of the scale reviewed the content of the translated versions of the Doloplus-2 [21–23].

To consider ‘hypothesis testing’, one study examined the correlations between the Doloplus-2 and the so-called ‘known correlates of pain’. This study found a statistically significant correlation between the Doloplus-2 and functional ability and depression in dementia [22]. Another study reported that there was no statistically significant difference between mean scores on the Doloplus-2 facial items across different levels of pain intensity [51]. A Known-groups technique was used to compare the Doloplus-2 scores of a ‘no pain’ group and a ‘daily pain’ group. This study found that the mean score was obviously higher in the ‘daily pain’ group than in the ‘no pain’ group. Another study reported low correlations between the Doloplus-2 and other measures of pain (the Pain Assessment Checklist for Seniors with Limited Ability to Communicate, the Pain Assessment in Advanced Dementia, the Visual Analogue Scale (VAS) and the Verbal Rating Scale) [24]. However, it is possible that self-rated pain, hypnotized correlates and other observational measures of pain, assess different dimensions of pain than the Doloplus-2 [22, 48]. One study reported that several items on the Doloplus-2 are related to delirium, depression and/or the severity of dementia; item 10 (‘Problems of behaviour’) on the psychosocial subscale appears to be the least specific [46].

##### Criterion validity

Five studies reported on the correlation between the Doloplus-2 and a ‘gold standard’ or ‘pain criterion’ [33, 42, 48, 49, 51]. A moderately high correlation (Spearman 0.7) was reported for the University of Alabama Birmingham Pain Behaviour Scale [33]. One study reported a low correlation (Pearson 0.4) with RNs’ yes/no rating of patient pain [48], and another study found that significantly more patients were evaluated as experiencing pain when using Doloplus-2 than with RNs’ proxy rating of pain [42]. No significant correlations were observed between the Doloplus-2 and the Facial Action Coding System at any level of pain intensity (mild, moderate or severe) [51].

One study reported a low correlation (Spearman 0.46) with patients’ self-assessment (VAS), but the correlation was higher in patients without dementia than in patients with dementia. Moreover, the Doloplus-2 predicted 41% of the variability in pain intensity as measured by the VAS where the somatic dimension explained the most [49]. Two studies compared the Doloplus-2 to experts’ pain ratings on the Numeric Rating Scale (NRS)-11. One found that the criterion validity of the Doloplus-2 was satisfactory and that the Doloplus-2 explained 62% of the experts’ pain score; the item ‘facial expression’ alone explained 48% of the experts’ scores [21]. The second study that used pain experts found no association between the experts’ ratings and the Doloplus-2 scores [47]. However, in this study, the criterion validity increased when the Doloplus-2 was administrated by a specialized geriatric nurse [47].

#### Responsiveness

Four studies examined the ability of the Doloplus-2 to detect changes in pain over time [53–56]. One study reported a statistically significant reduction in the total mean score after one year of monthly assessments [53], while three studies demonstrated a statistically significant reduction in the total [54–56] and subscale scores [55] post-treatment.

## Discussion

This review synthesizes the available research on the feasibility, clinical utility and measurement properties of the Doloplus-2 pain scale in older adults with cognitive impairment. Previous reviews have concluded that there is limited evidence for the feasibility, clinical utility, and validity of the measurement properties of pain assessment tools for older adults with cognitive impairment [9, 15]. Based on the 24 studies summarized in this review, we draw a similar conclusion for the Doloplus-2. Of the studies evaluated, only four studies were assessed as high-quality studies based on the MMAT. There were significant variations in the designs and methods of analysis in the included studies. The majority were performed in LTC settings with patients with cognitive impairment and used small, heterogeneous samples, which limited the possibility of sub-group analyses. Consequently, it is difficult to draw conclusions about the suitability and effectiveness of the scale in various subpopulations (i.e. varying types and degrees of cognitive impairment). Furthermore, the methods of assessing pain with the Doloplus-2 varied across the studies. There was considerable variation in how the studies reporting on at least one of the COSMIN measurement properties assessed reliability, validity and responsiveness. Likewise for the handful of studies that explicitly assessed feasibility and clinical utility, which also used small samples.

Because older adults with cognitive impairment (especially in the severe stage) often have a limited ability to communicate pain, their expressions of pain may not be obvious and may be difficult to interpret. Consequently, it is essential that clinicians and researchers use appropriate, effective tools when assessing pain in older adults with cognitive impairment. Furthermore, the measurement properties of such tools are not fixed attributes of the scale and vary according to population [57, 58], and validation is a long process which needs to be repeated [47, 59]. These findings have several implications for clinical practice and future research.

First, it must be further evaluated whether and how the results of the Doloplus-2 assessment can guide clinical decisions and improve patient outcomes. This may vary across settings and populations. One important issue is whether all of the Doloplus-2 items detect pain, rather than other symptoms, in older adults with cognitive impairment [21, 22, 24, 46]. The overlap between manifestations of pain and those of delirium, dementia and/or depressive symptoms can make it difficult to assess and confidently identify pain (distinct from delirium or depressives symptoms) in this population, who are prone to these comorbidities [60, 61]. This may affect treatment decisions based on Doloplus-2 assessments and the quality of the pain management. Previous studies have reported that nurses and physicians experience some uncertainty about the accuracy of pain assessment in older adults with cognitive impairment, and they may be reluctant to administer analgesics as a result of this uncertainty [8]. A combination of Doloplus-2 assessment with the use of observational tools to evaluate comorbidities such as depressive symptoms and delirium may increase the scale’s validity and its ability to provide significant clinical information about pain in this population.

The Doloplus-2 is one of the few observational pain assessment tools that provides a cut-off to categorize patients with ‘pain’ and ‘no pain’ [9]. The developers of the Doloplus-2 recommend a cut-off ≥5, but they also point out that pain cannot be excluded even with a score below 5 [17, 19]. A cut-off score can make the results of the assessment easier to interpret and more meaningful and actionable [58, 62] in clinical practice and research. To our knowledge, this cut-off, which is based on clinical experience [19], has not been evaluated. Questions have been raised about whether the established cut-off will entail an under- or overestimation of pain [43, 49]. According to the Doloplus-2 Group, higher scores indicate increasing pain intensity [19]. However, there is no evidence supporting the assumption that HCPs can determine pain intensity from patient behaviour [15], nor is there evidence suggesting that it is appropriate to assume that intensity of behaviour is proportional to intensity of pain. Therefore, we argue that the Doloplus-2 only indicates whether a patient may be in pain or not; it does not indicate anything about the intensity of the patient’s pain. Thus, there is a need to validate the cut-off score and to examine HCPs’ interpretations of the (change in) score. How the score informs clinical decisions and actions must also be evaluated, as this is an important indication of the scales’ clinical utility in everyday practice.

Second, more research is needed concerning the feasibility of the Doloplus-2 across settings and populations. There appear to be large variations in how the Doloplus-2 is administered. These variations include the raters’ professional qualifications, the training provided (if any), and raters’ familiarity with the patients’ usual behaviour and habits. As the developers of the Doloplus-2 point out, using the scale requires training [17]. The raters need to understand how it works and the terminology used in the scale. Use of the scale also requires an ability to note changes in a patient’s usual behaviour and an awareness of pain and pain control in older adults not able to self-report pain [17, 19] in order to plausibly achieve the best fit between the rater’s assessment and the patient’s experience [9].

However, while such an ideal situation might be feasible for a research study, is it feasible for everyday clinical use? Providing training and securing the availability of staff familiar with patients demands many resources and may impede the scales’ feasibility. Across health care settings, staff turnover is high and changing work shifts are common. Furthermore, a shortage of nurses is projected in the next 10 to 20 years [63]. Therefore, the most realistic scenario involves a care facility with a significant number of HCPs who have varying amounts of training, professional and personal skills, and familiarity with the patients administering the scale, which may affect its reliability [38].

The administration, scoring and interpretation of the scale also needs to be described in an unambiguous, reproducible manner. According to the Doloplus-2 guidelines, items on the scale should not be scored if they do not apply to the patient [17]. This is a methodological concern because the total score is affected by unanswered items. It is not clear whether a minimum number of items must be answered in order to use the scale correctly [54]. Consequently, if the Doloplus-2 is to be used in everyday clinical practice, it may be necessary to evaluate the scales’ guidelines and determine what actually works in the variety of settings where older adults with cognitive impairment receive health care. Furthermore, how to effectively and easily facilitate everyday use while obtaining valid, reliable results should be explored.

Third, the Doloplus-2 is based on sound assumptions about the multidimensionality of pain. Its items are supported by the literature on how older adults who are unable to communicate verbally express pain [15]. However, the results of our review suggest that there is limited research on the validity of the content of the Doloplus-2. No studies have been done to determine whether clinicians and experts in the various fields of caring for older adults with different types and stages of cognitive impairment consider the scale to be comprehensive. As previously discussed, some items of the Doloplus-2 have been reported to be difficult to administer, probably because the items are somewhat unspecific regarding pain, which may lead to uncertain results. Even though face validity only provides information about whether the Doloplus-2 appears to measure pain, it is still important, as clinicians and experts need to have confidence in the scales’ relevance to the construct they want to measure.

Furthermore, it is necessary to evaluate whether the items are equivalent in all multilingual versions, and whether all translated versions of the Doloplus-2 are conceptually, semantically and operationally equivalent [58] to the original French version. If different versions of the Doloplus-2 are not equivalent, it is uncertain whether observed differences in, for example, pain prevalence assessed with the Doloplus-2 are due to actual differences in pain or subtle variations in what the tool is actually measuring. Comparing results and interpreting differences or similarities must be done with caution [58]. Additionally, translation issues, such as ambiguous wording that different raters may understand differently, may lead to inconsistency in scoring some items [21].

The results of our review suggest that it is difficult to establish the construct and criterion validity of the Doloplus-2. The studies included in this review used a variety of hypothesized pain criteria and pain correlates (measures for the same/unrelated constructs) to test these aspects of the scale’s validity. Moreover, tests were conducted under a wide range of circumstances and samples. There is no gold standard to use as a benchmark for the assessment of pain in older adults with cognitive impairment due to the subjectivity of pain, and that makes it difficult to evaluate the scale’s criterion validity [9].

There is also a lack of interventional studies using rigorous investigation methods, and there is limited evidence regarding the responsiveness of the Doloplus-2. An unresponsive instrument may indicate an improvement in the patient’s pain when there actually is none, or it may fail to detect true improvement. There is some controversy over trying to test ‘responsiveness’ as a property of an instrument as it is hard to disentangle the instrument’s characteristics from the characteristics of the treatment provided [58]. However, it is important for clinicians and researchers to know if an intervention induces change in the patient’s condition. Therefore, future research should investigate whether the Doloplus-2 measures change in a meaningful way and whether it can be used to evaluate the effect of pain treatments in older adults with cognitive impairment.

### Strengths and limitations

This review has several strengths. We used systematic methods and multiple sources to identify relevant studies. We also included articles written in other languages than English. Two reviewers independently assessed the titles, abstracts and quality of the studies. The MMAT was used for quality assessment to allow for the different study designs included in this review, and, in order to provide a comprehensive review, studies were not excluded based on methodological quality. Two reviewers independently abstracted data according to the COSMIN guidelines; this meant that measurement properties were assessed in a uniform way to avoid confusion regarding relevance, terminology, definitions and design.

One limitation of this review is that the authors of the included studies may have used different definitions for the measurement properties than those provided by COSMIN, which may have led us to misinterpret or misrepresent their findings. An example provided by the COSMIN initiative is the definition of ‘responsiveness’, which may be defined as “the ability to detect clinically important change” or as “the ability to detect change in the construct to be measured”. These definitions reflect different constructs [31].

Furthermore, our findings are limited due to the heterogeneity of the included studies. Also, some quality criteria of included studies may have been rated as insufficient simply because the necessary information was not available. Four studies that may have had important findings were excluded because we were unsure whether they fulfilled the inclusion criteria. Although we tried to contact the authors of these articles, we were unsuccessful, which may be due to the fact that some of these studies were published ten to fifteen years ago. Finally, approximately one-third of our included studies were retrieved from the supplementary sources. This might indicate a possible bias in the systematic search strategy in the databases, such as missing indexed terms, possibly resulting in a lower number of articles and thereby incomplete conclusions.

Despite these limitations, our review is relevant for both clinicians and researchers. It provides valuable insight about the evidence regarding aspects of the use and the measurement properties of the Doloplus-2. It also highlights some of the complex, challenging issues in the field of pain assessment in older adults with cognitive impairment.

## Conclusion

The Doloplus-2 has been cited as one of the more extensively tested and promising tools for pain assessment in older adults with cognitive impairment. Still, this review suggests that there is a lack of comprehensive, high-quality evidence regarding the feasibility, clinical utility and measurement properties of this scale when assessing pain in older adults with cognitive impairment. Further research should examine the Doloplus-2 across a range of settings. Moreover, future studies should use more homogenous samples and provide clear definitions of the type and stage of cognitive impairment and pain. Also, more studies should be done using rigorous methods and large sample sizes in order to better allow clinicians and researcher to assess the tools’ effectiveness and appropriateness for measuring pain in older people with cognitive impairment.

## Additional files

Additional file 1: Search Strategy as used in CINAHL. (DOCX 12 kb)

Additional file 2: Quality assessment using the Mixed Methods Appraisal Tool. (DOCX 20 kb)

## Abbreviations

COSMIN: COnsensus-based Standards for the selection of health Measurement INstruments
HCP: Health Care Providers
ICC: Intraclass Correlation Coefficient
LTC: Long-term care
MMAT: Mixed Methods Appraisal Tool
NA: Nursing Assistant
RA: Research Assistant
RN: Registered Nurse
VAS: the Visual Analogue Scale
