# Supplementary material for: Measurement properties, feasibility and clinical utility of the Doloplus-2 pain scale in older adults with cognitive impairment: a systematic review
Source: BMC Geriatr. 2017 Nov 2;17:257. doi: 10.1186/s12877-017-0643-9 (PMC5667437; doi:10.1186/s12877-017-0643-9)
Supplement: Supplementary file 1 — Search Strategy as used in CINAHL. (DOCX 12 kb) [file 12877_2017_643_MOESM1_ESM.docx]

| **# Searches** |
| --- |

1 (MH "Cognition Disorders") OR (MH "Cognition")

2 cogniti*

3 S1 OR S2

4 (MH "Dementia+")

5 dement* or alzheimer*

6 dement* or alzheimer*

7 (MH "Pain+")

8 pain or discomfort

9 S7 OR S8

10 (MH "Pain Measurement")

11 assess* or evaluat* or measur* or validat*

12 S10 OR S11

13 (MH "Instrument by Type") OR (MH "Research Instruments") OR (MH "Clinical Assessment Tools") OR (MH "Severity of Illness Indices") OR (MH "Health Status Indicators") OR (MH "Questionnaires+") OR (MH "Scales") OR (MH "Checklists") OR (MH "Behavior Rating Scales")

14 instrument* or scale* or question* or tool or tools or checklist*

15 S13 OR S14

16 S6 AND S9 AND S12 AND S15

17 S3 AND S9 AND S12 AND S15

18 S1 AND S9 AND S12 AND S15

19 S16 OR S18

20 S16 OR S18 Limiters - Published Date: 19900101-; Language: Danish, Dutch/Flemish, English, French, German, Norwegian, Swedish

21 (MH "Aged") OR (MH "Aged, 80 and Over")

22 old or aged or elderly or adult* or older or senior* or veteran*

23 S21 OR S22

24 S20 AND S23

|  |
| --- |
