# Supplementary material for: Measurement properties, feasibility and clinical utility of the Doloplus-2 pain scale in older adults with cognitive impairment: a systematic review
Source: BMC Geriatr. 2017 Nov 2;17:257. doi: 10.1186/s12877-017-0643-9 (PMC5667437; doi:10.1186/s12877-017-0643-9)
Supplement: Supplementary file 2 — Quality assessment using the Mixed Methods Appraisal Tool. (DOCX 20 kb) [file 12877_2017_643_MOESM2_ESM.docx]

**Additional file 2a** Quantitative descriptive studies

| **First author, year, [reference]** | **Screening questions** |  | **Quantitative descriptive studies** |  |  |  |
| --- | --- | --- | --- | --- | --- | --- |
|  | A. Are there clear qualitative and quantitative research questions (or objectives), or a clear mixed methods question (or objective)? | B. Do the collected data allow address the research question (objective)? | 4.1. Is the sampling strategy relevant to address the quantitative research question (quantitative aspect of the mixed methods question)? | 4.2. Is the sample representative of the population under study? | 4.3. Are measurements appropriate (clear origin, or validity known, or standard instrument)? | 4.4. Is there an acceptable response rate (60% or above)?* |
| Akbarzadeh, 2007, Sweden [23] | Yes | Yes | Yes | Can’t tell | Yes | Can’t tell |
| Ando, 2010, Japan [52] | Yes | Yes | Yes | Can’t tell | Yes | Can’t tell |
| Bauer, 2007, France [36] | Yes | Yes | Yes | Can’t tell | Yes | Can’t tell |
| Chen, 2010a, Taiwan [51] | Yes | Yes | Yes | Can’t tell | Yes | Yes |
| Chen, 2010b, Taiwan [50] | Yes | Yes | Yes | Can’t tell | Yes | Can’t tell |
| Chen, 2014, Taiwan [49] | Yes | Yes | Yes | Can’t tell | Yes | Yes |
| Couilliot, 2013, France [37] | Yes | Yes | Yes | Yes | Yes | Yes |
| Hadjistavropolous, 2008, Canada [43] | Yes | Yes | Yes | Can’t tell | Yes | No, only 54.6% of the approached patients agreed to participate, and no information about those who refused are reported |
| Hølen, 2005, Norway [32] | Yes | Yes | Yes | Can’t tell | Yes | Can’t tell |
| Hølen, 2007, Norway [33] | Yes | Yes | Yes | Can’t tell | Yes | Can’t tell |
| Monacelli, 2013, Italy [42] | Yes | Yes | Yes | Yes | Yes | Yes |
| Neville, 2014, Australia [48] | Yes | Yes | Yes | Can’t tell | Yes | Can’t tell |
| Pautex, 2007, Switzerland [39] | Yes | Yes | Yes | Can’t tell | Yes | Can’t tell |
| Pickering, 2010, multinational [54] | Yes | Yes | Yes | Can’t tell | Yes | Can’t tell |
| Sheu, 2011, Canada [47] | Yes | Yes | Yes | Can’t tell | Yes | Can’t tell |
| Stacpoole, 2014, UK [40] | Yes | Yes | Yes | Can’t tell | Yes | Can’t tell |
| Torvik, 2009, Norway [35] | Yes | Yes | Yes | Yes | Yes | Yes |
| Torvik, 2010, Norway [34] | Yes | Yes | Yes | Yes | Yes | No, some of the approached patients refused to participate, and some of the enrolled patients had incomplete assessments, leaving 57% |
| Voyer, 2008, Canada [46] | Yes | Yes | Yes | Can’t tell | Yes | No, only 54.6% of the approached patients agreed to participate, and no information about those who refused are reported |
| Voyer, 2009, Canada [44] | Yes | Yes | Yes | Can’t tell | Yes | No, only 54.6% of the approached patients agreed to participate, and no information about those who refused are reported |
| Voyer, 2011, Canada [45] | Yes | Yes | Yes | Can’t tell | Yes | Can’t tell |
| Zwakhalen, 2006, the Netherlands [41] | Yes | Yes | Yes | Can’t tell | Yes | Can’t tell |

* Response rate was assessed as both number asked to participate vs number entered into the study AND the number of patients participating vs the number of patients sampled

**Additional file 2b** Quantiative randomized controlled (trials) and Quantitative non-randomized studies

| **First author, year, [reference]** | **Screening questions** |  | **Quantitative randomized controlled (trials)** |  |  |  | **Quantitative non-randomized** |  |  |  |
| --- | --- | --- | --- | --- | --- | --- | --- | --- | --- | --- |
|  | A. Are there clear qualitative and quantitative research questions (or objectives), or a clear mixed methods question (or objective)? | B. Do the collected data allow address the research question (objective)? | 2.1. Is there a clear description of the randomization (or an appropriate sequence generation)? | 2.2. Is there a clear description of the allocation concealment (or blinding when applicable)? | 2.3. Are there complete outcome data (80% or above)? | 2.4. Is there low withdrawal/drop-out (below 20%)? | 3.1. Are participant (organizations) recruited in a way that minimizes selection bias? | 3.2. Are measurements appropriate (clear origin, or validity known, or standard instrument; and absence of contamination between groups when appropriate) regarding the exposure/intervention and outcomes? | 3.3. In the groups being compared are the participants comparable, or do researchers take into account (control for) the difference between these groups? | 3.4. Are there complete outcome data (80% or above), and, when applicable, an acceptable response rate (60% or above), or an acceptable follow-up rate for cohort studies |
| Ando, 2016, Japan [53] | Yes | Yes | Yes | Yes | Yes | Can’t tell |  |  |  |  |
| Rodríguez-Mansilla, 2015, Spain [38] |  |  |  |  |  |  | Yes | Yes | Yes | Yes |
